# Transforming Waste into Value: A Sustainable Zero-Waste Biorefinery for Biochar Production and Gallic Acid Adsorption from Apple Pomace

**DOI:** 10.1021/acsomega.5c06957

**Published:** 2025-10-17

**Authors:** Josiel Martins Costa, Leda Maria Saragiotto Colpini, Tânia Forster-Carneiro

**Affiliations:** † Faculdade de Engenharia de Alimentos (FEA), 28132 Universidade Estadual de Campinas (UNICAMP), Rua Monteiro Lobato, 80, 13083-862 Campinas, São Paulo, Brazil; ‡ Graduate Program in Technology and Environmental Engineering, Federal University of Parana (UFPR), Rua Pioneiro, 2153, 85953-128 Palotina, Paraná, Brazil

## Abstract

The zero-waste biorefinery maximizes the use of biomass and reduces environmental impacts, transforming waste into high value-added products. In this study, the apple pomace underwent three rounds of extraction of phenolic compounds, sugar, and pectin recovery. Considering the biorefinery concept, the solid residue from both processes was used for biochar synthesis. The evaluation of the adsorptive efficiency of biochar and its characterization occurred by three synthesis routes: (1) biochar from the residue after the extraction of phenolic compounds and sugar (CPB); (2) from the residue after the extraction of phenolic compounds, sugar, and pectin (CPPB); and (3) from dry apple pomace (APB). The sequential extraction yields of total phenolic compounds were 4.6 ± 0.2, 1.2 ± 0.1, and 0.33 ± 0.01 mg GAE g^–1^, respectively, for the first, second, and third extraction rounds. The pectin yield was 13.74 ± 0.25% with a degree of esterification of 66.38%. The gallic acid adsorption assay at 10 mg L^–1^ provided an adsorption efficiency of 89.93 ± 0.87% for the CPPB sample. The Avrami model with a theoretical equilibrium adsorption of 7.7 ± 0.1 mg g^–1^ biochar represented the adsorption kinetics of gallic acid by the CPPB sample with *R* ^2^ = 0.9953. The intraparticle diffusion model presented multilinearity with two stages. Finally, the Path2green sustainable extraction metric scored 0.665, demonstrating strong adherence to green chemistry principles and new perspectives for industrial processes applied to the pharmaceutical, food, and cosmetic sectors.

## Graphical abstract

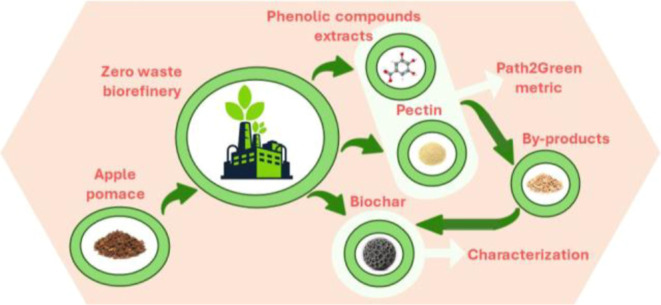

## 1 Introduction

The generation of organic solid waste has become a global challenge due to the increased food production and mass consumption. The agri-food industry generates large volumes of waste, which are often disposed of inappropriately, contributing to adverse environmental impacts, such as greenhouse gas emissions and soil and water contamination.^1,2^ In addition, the waste of the byproducts represents a significant loss of resources that would positively affect human and animal health.^3^ Therefore, searching for sustainable strategies is essential to promoting a resource-efficient economy and reducing the environmental impacts of inappropriate disposal.

Apple pomace is a byproduct of the juice and fruit processing industry, representing approximately 25–30% of the total weight of the processed apple.^4^ This byproduct comprises insoluble fibers like cellulose, hemicellulose, lignin and pectin, sugar, and a significant fraction of bioactive compounds, including phenolic compounds and antioxidants. The phenolic compounds in apple pomace have health-benefiting properties, such as antioxidant, anti-inflammatory, anticancer, and antimicrobial actions, making them valuable ingredients for the pharmaceutical and food industries.^5,6^ In addition, pectin, a structural polysaccharide abundant in apple pomace, is widely used as a gelling and stabilizing agent in the food industry^7^ and has biomedical and pharmaceutical applications. Furthermore, apple pomace has energy potential for producing methane-rich biogas through the conversion of organic matter by the microbial consortium during anaerobic digestion.^8^

Currently, the predominant destination for apple pomace includes animal feed, composting, and, in some cases, landfill disposal. Although these options can partially reduce the environmental impact, they do not fully consider the potential of the residue as a source of valuable biocompounds. Animal feed, for example, underutilizes high value-added compounds, such as polyphenolic compounds like cinnamic acid, epicatechin, caffeic acid, and procyanidin,^9^ in addition to sugar and pectin. Landfill disposal, in addition to representing a waste of resources, contributes to the emission of greenhouse gases due to the anaerobic decomposition of organic matter.^10^ Therefore, the valorization of apple pomace should be conducted by exploiting its compounds of commercial interest.

In recent years, apple pomace has attracted increasing attention for valorization due to its abundance, composition, and versatile applications across multiple sectors. It has been investigated as a source of bioactive compounds such as phenolics, flavonoids, and triterpenoids.^11–13^ The extraction of these compounds adds economic value and reduces the environmental burden associated with conventional disposal. Moreover, apple pomace has been explored as a substrate for fermentable sugars, enabling conversion into bioethanol and other biochemicals and advancing the development of sustainable biobased industries.^14,15^

Despite increasing interest in the valorization of apple pomace, most studies remain limited to single applications without integrating multiple pathways. In general, sequential extraction strategies coupled with the conversion of residual biomass into high-value materials have been overlooked, leaving the final solid residue to be underutilized. Implementing such integrated strategies aligns with circular economy principles, promoting zero-waste management and maximizing the utilization of apple pomace. The extraction of compounds provides interesting products, such as vitamins C and E,^16^ flavonoids,^17^ triterpenoids,^18^ phytosterols,^19^ dietary fiber,^20^ ursolic acid,^21^ amino acids,^22^ and fermentable sugar.^23^ Furthermore, the residual biomass obtained after extraction can be converted to biochar, a carbon-rich material of interest for both environmental and energy applications. Beyond serving as an adsorbent for pollutants or as a renewable energy source,^24^ biochar is increasingly recognized as a sustainable and low-cost porous carbon with diverse uses, including electrode development,^25^ soil remediation,^26^ and greenhouse gas mitigation.^27^

Zhang et al.^28^ synthesized magnetic biochar from apple pomace by pyrolysis at 600 °C, followed by immersion aging in Fe­(II)/Fe­(III) aqueous solution. The authors highlighted that batch adsorption provided a maximum adsorption capacity of 818.4 mg g^–1^ of Ag­(I), with intraparticle diffusion being the presumed adsorption mechanism. Additionally, column adsorption tests demonstrated that biochar could enrich and separate Ag­(I) from the aqueous system. Although other studies involving biochar from apple pomace have been reported,^29,30^ none have addressed sequential extractions prior to the use of the residue. Thus, the implementation of an integrated biorefinery approach allows the full use of the residue, in line with the principles of circular economy and the bioeconomy. Clean and circular production is emerging as a key driver of next-generation manufacturing across industries.^31^

Based on this approach, the study sought to valorize apple pomace through a zero-waste biorefinery approach, aiming at the sequential extraction of phenolic compounds, sugar, and pectin and the synthesis of biochar from the final solid residue. This study focuses on biochar due to its stability, high carbon content, and adsorption capacity, its integration potential within a biorefinery framework, and its suitability for demonstrating environmental applications in phenolic compound adsorption. The adsorbent capacity of the biochar was compared with commercial adsorbents and evaluated considering three scenarios: (1) after the extraction of phenolic compounds and sugar in three rounds; (2) after the extraction of phenolic compounds and sugar in three rounds and pectin extraction; and (3) from dry apple pomace.

## 2 Materials and Methods

### 2.1 Extraction of Phenolic Compounds and Pectin

The chemicals and raw material used in all experiments are described in Supporting Information Section 1. Figure 1 shows the flowchart of the apple pomace biorefinery, considering the extraction of phenolic compounds, pectin, and biochar synthesis from three routes. The apple pomace was initially ground in a blender (Waring, model MX1500). The average particle size, lipid content, moisture content, total fixed solids, and total volatile solids were determined, as done previously.^32^ The phenolic compounds were extracted in three rounds using 20 g of apple pomace and a solvent/feed ratio of 10:1, with 50% w/w ethanol solution. Each round of extraction occurred at 60 °C under agitation for 30 min. After this, the samples were centrifuged at 4000 rpm using a centrifuge. The extracts were collected and stored. The solid residue was dried in an oven at 105 °C for subsequent biochar production.

**Figure 1:**
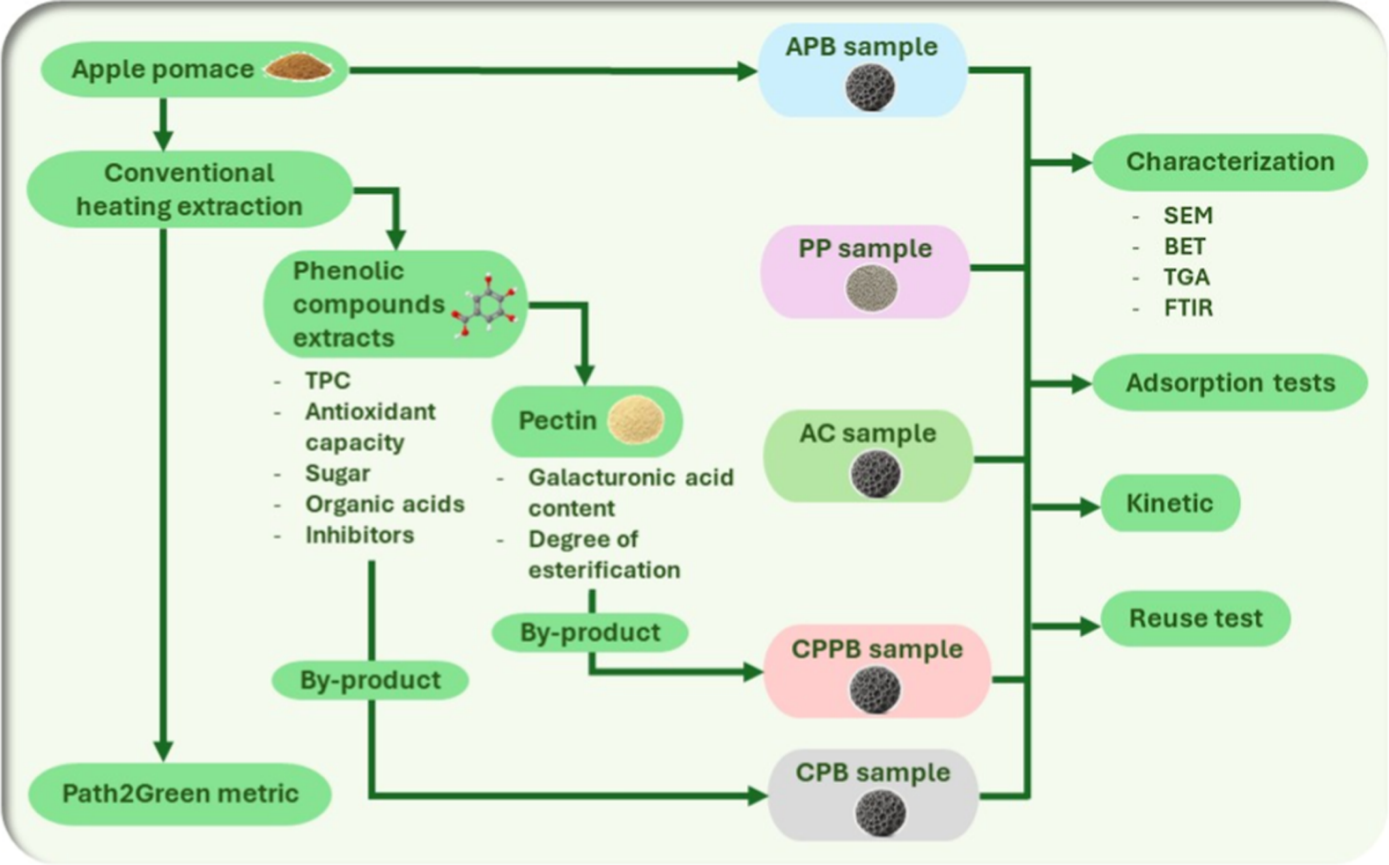
Flowchart of the apple pomace biorefinery for biochar synthesis from three routes: (1) from dry apple pomace—APB sample; (2) after the extraction of phenolic compounds and sugar in three rounds and pectin extraction—CPPB sample; and (3) after the extraction of phenolic compounds and sugar in three rounds—CPB sample.

For pectin extraction, the phenolic compounds were extracted as previously described. After centrifugation of the samples, a citric acid solution (6.5% w/v) pH 2.0 in a solvent/feed ratio of 10:1 was added to the solid residue. The solution was kept at 90 °C under stirring for 90 min. The samples were centrifuged at 4000 rpm using a centrifuge. Pectin precipitation occurred in a 1:1 (v/v) ratio with ethanol by using the supernatant gel. The solid residue was dried in an oven at 105 °C for subsequent biochar production. Eq 1 describes the pectin yield (%).

1 $$Pectin\;yield\left(\%\right)=\frac{dry\;pectin\;mass\;\left(g\right)}{dry\;apple\;pomace\;mass\;\left(g\right)}\times 100$$

### 2.2 Biochar Synthesis

Biochar synthesis occurred from dried apple pomace (APB) and after the extraction of phenolic compounds and sugar (CPB) and phenolic compounds, sugar, and pectin (CPPB). Additional information regarding the description of the procedure can be found in Supporting Information Section 1.

### 2.3 Characterization of the Extracts throughout the Determination of Total Phenolic Compounds, Sugar, Organic Acids, Inhibitors, and Antioxidant Capacity

The total phenolic compound (TPC) content of the extracts considering the three rounds of extraction was determined by the Folin–Ciocalteu colorimetric method, as described by Silva et al.^33^ The analysis of sugars, organic acids, and inhibitors was performed by high-performance liquid chromatography (HPLC) with a refractive index detector (RID), following the method described by Barroso et al.^34^ The antioxidant capacity was assessed using the Ferric Reducing Antioxidant Power (FRAP) assay, following the protocol described by Silva et al.,^33^ with minor modifications. Galacturonic acid content was determined according to the study of Pereira et al.,^35^ with minor modifications. Additional information regarding the description of the procedure can be found in Supporting Information Section 1.

### 2.4 Gallic Acid Adsorption Tests

The gallic acid adsorption tests were conducted at concentrations of 10, 50, 250, and 500 mg L^–1^. The tests used 10 mL of gallic acid solution with an adsorbent concentration of 1 g L^–1^ (*m* = 0.01 g), pH 3.5, and a reaction time of 22 h under slow stirring at room temperature. The adsorptive capacity was evaluated by removing gallic acid from the solution using a UV–vis spectrophotometer at a wavelength of 760 nm and a calibration curve, as described in Supporting Information Section 1. The biochars produced, APB, CPB, and CPPB, in addition to porapak (PP) and commercial activated carbon (AC), were considered for duplicate tests. Eq 2 describes the adsorption efficiency of the biochar:

2 $$R\left(\%\right)=\left(\frac{C_{0}-C}{C_{0}}\right)\times 100$$

where *R*—gallic acid adsorption efficiency from the solution (%), *C*_0_—initial concentration of gallic acid (mg L^–1^), and *C*—final concentration of gallic acid (mg L^–1^).

### 2.5 Gallic Acid Adsorption Kinetics and Biochar Reuse Tests

The adsorption kinetics were performed for the biochar produced from apple pomace with a higher adsorption efficiency to determine the equilibrium time. The tests were conducted using 15 mL of 10 mg L^–1^ gallic acid solution, adsorbent concentration of 1 g L^–1^, at room temperature, and under stirring. Aliquots of 300 μL were collected at intervals of 5 min (from 0 to 15 min), 15 min (from 15 to 120 min), and 1 h until reaching equilibrium. Eq 3 determined the adsorbed amount of gallic acid (*q*_*t*_) on the biochar at time *t*:

3 $$q_{t}=\left(\frac{C_{0}-C_{t}}{m}\right)V$$

where *q*_*t*_—amount of gallic acid adsorbed on biochar at time *t* (mg gallic acid g^–1^ biochar); *C*_0_—initial concentration of gallic acid (mg L^–1^); *C*_t_—final concentration of gallic acid at time *t* (mg L^–1^); *m*—mass of biochar (g); and *V*—volume of gallic acid solution (L).

The theoretical equilibrium adsorption of gallic acid (*q*_e_) was estimated using the pseudo-first-order, pseudo-second-order, and Avrami and Elovich kinetic models. The mass transfer mechanism of gallic acid within the biochar considered the intraparticle diffusion model.

The biochar reuse tests were conducted with a 10 mg L^–1^ gallic acid solution, pH 3.5, and a biosorbent concentration of 1 g L^–1^, at room temperature, under stirring for 3.5 h. The desorption of gallic acid from biochar occurred with 70% v/v ethanol solution, at room temperature, under stirring for 3.5 h. Aliquots were collected at the end of adsorption and desorption to determine the gallic acid content, as described in Supporting Information Section 1. The data were expressed as q_t_ values.

### 2.6 Biochar Characterization

The morphologies of the biochar samples (APB, CPB, and CPPB), PP, and AC were characterized before and after the adsorption of gallic acid (10 mg L^–1^) using scanning electron microscopy (SEM) and energy-dispersive spectroscopy (EDS). The samples were coated with gold, and the images were magnified 500 and 1000 times. The EDS technique determined the elemental composition of the samples.

The identification of functional groups of the samples was performed using a Fourier transform infrared (FTIR) spectrophotometer with a wavelength of 4000–600 cm^–1^. The pore size distribution was performed by the Barrett–Joyner–Halenda (BJH) method through N_2_ adsorption–desorption isotherms using a surface area analyzer (NOVA 2000e—Quantachrome Instruments). The surface area, pore volume, and pore diameter were determined by the Brunauer–Emmett–Teller (BET) method.

The thermal behavior of the samples was evaluated by thermogravimetric analysis (TGA) using a thermogravimetric analyzer (PerkinElmer, model STA 6000, Akron, USA). The sample was heated to 105 °C and kept at this temperature for 5 min to completely remove moisture. After that, the sample was heated to 900 °C at a heating rate of 10 °C min^–1^ under a nitrogen atmosphere. The results were expressed as the percentage of mass loss as a function of the temperature increase. In addition, TGA was performed with apple pomace to determine the lignocellulosic composition of the residue. The quantification of the lignocellulosic components of apple pomace was based on the derived thermogravimetric (DTG) analysis. The decomposition peaks in the DTG curves were identified to attribute mass losses corresponding to specific components: 200–300 °C for hemicellulose, 300–400 °C for cellulose, and 400–600 °C for lignin, as reported by Díez et al.,^36^ Xiao et al.,^37^ Carrier et al.,^38^ and Chen et al.^39^ The mass fractions of cellulose, hemicellulose, and lignin were calculated according to the method described by Aguilar–Aguilar et al.^40^

### 2.7 Statistical Analysis

The statistical analysis considered the Tukey test to compare the means. The analysis of kinetic and equilibrium data used nonlinear techniques (Simplex method and Levenberg–Marquardt algorithm) through the OriginPro 2016 software.

### 2.8 Sustainability Assessment of the Apple Pomace Biorefinery Using the Path2Green Metric

The extraction of phenolic compounds, sugar, and pectin and biochar production were assessed using the Path2Green metric.^41^ Supporting Information Section 1 provides additional information regarding the description of the procedure.

## 3 Results and Discussion

### 3.1 Total Phenolic Compounds, Sugar Content, and Pectin

According to Table 1, the sequential extraction of TPC from apple pomace provided concentrations of 4.6 ± 0.2, 1.2 ± 0.1, and 0.33 ± 0.02 mg GAE g^–1^, respectively, for the first, second, and third extraction rounds. The multiple-round extraction was a strategy to maximize the full utilization of the plant matrix regarding the TPC of interest due to their applications in metabolic disorders such as diabetes^42^ and their antimicrobial properties.^43^ The extraction in more than one round aimed to minimize the following negative effects: (1) uneven distribution of phenolic compounds in the cellular compartments of the plant matrix; (2) solvent saturation upon reaching its solubilization limit; (3) limited diffusion of components in the liquid phase; (4) matrix heterogeneity caused by different particle sizes and porosities; and (5) chemical interactions of phenolic compounds that lead to the formation of complexes with proteins and polysaccharides.

**Table 1:** TPC, Antioxidant Activity, Sugar, Inhibitor, and Organic Acid Content of the Extraction in Three Rounds

compound	1° round	2° round	3° round
TPC (mg GAE g^–1^)	4.6 ± 0.2	1.2 ± 0.1	0.33 ± 0.01
FRAP (μmol TE g^–1^)	192 ± 4	45 ± 2	14.1 ± 0.5
acetic acid (mg g^–1^)	n.d.	n.d.	n.d.
glucose (mg g^–1^)	97.5 ± 1.7	24.3 ± 0.8	4.8 ± 0.4
fructose (mg g^–1^)	265 ± 8	73.2 ± 0.2	13.7 ± 1.1
5-HMF (mg g^–1^)	n.d.	n.d.	n.d.

Du et al.^44^ reported a TPC content of 2228.49 ± 66.78 mg GAE L^–1^ in apple peels by conventional extraction at 30 °C for 30 min in a shaker, using 70% methanol containing 2% formic acid, separately, both as extracting solvents. On the other hand, apple pulp presented a content of 208.75 ± 9.28 mg GAE L^–1^. Compared to this study with the same dimensional unit, the sum of the 3 extraction rounds provided a TPC content of 611 ± 16 mg GAE L^–1^. Therefore, the content was between the values reported for the apple peel and pulp. In addition, ethanol as a solvent used in conventional extraction is a green, nontoxic solvent, compared to methanol. Thus, the applications of the extracts safely allow their wide use in various industrial sectors.

Antioxidant activity is generally attributed to the presence of individual phenolic compounds, often exhibiting a linear relationship with the total antioxidant capacity.^45^ Phenolics such as chlorogenic acid, gallic acid, catechins, and quercetin derivatives, commonly found in apple byproducts, possess redox properties that enable them to function as effective electron donors and metal chelators. The mechanisms are essential for neutralizing reactive oxygen species. From the first to the second extraction, the antioxidant potential decreased by approximately 4-fold, while a further 3-fold reduction was observed from the second to the third extraction. Linear regression analysis between TPC and antioxidant capacity revealed a coefficient of determination (*R* ^2^) of 0.99, indicating an almost perfect linear correlation. This strongly supports the hypothesis that phenolic compounds are the primary contributors to the antioxidant potential of the extract. Collectively, these findings underscore the critical role of phenolic constituents in shaping the antioxidant behavior of agro-industrial residues and support the development of functional ingredients derived from fruit processing byproducts.

As shown in Table 1, the extracts did not present inhibitors such as 5-HMF, due to the mild extraction temperature at 60 °C. Likewise, no organic acids, such as acetic acid, were detected. Figure S1 displays the chromatogram of the standards compared with a sample. The first round of extraction presented the highest concentration of fructose (265 ± 8 mg g^–1^) and glucose (97.5 ± 1.7 mg g^–1^), demonstrating the potential of apple pomace as an energy source, for example, as a substrate for fermentation and ethanol production. The second and third rounds of extraction led to concentrations of fructose and glucose approximately 4 to 5 times lower than the previous round, indicating that the multistage extraction optimized the yield by maximizing the concentration gradient between the solid and the liquid.

Pectin extraction after phenolic compound extraction provided a pectin yield of 13.74 ± 0.25%. Previous studies reported pectin yields of 25.27 ± 1.78% ^9,32^ and 5 ± 0.3%^4^ by conventional heating extraction and similar conditions. However, for the first system, precipitation and washing with ethanol occurred once. In the second system, pectin precipitation and washing three times led to the loss of soluble pectin fractions, especially lower-molecular-weight chains. Thus, the intermediate yield in this study was a consequence of pectin precipitation and washing in a single round and prior extraction of phenolic compounds in three rounds, which may have removed more soluble or structural pectin fractions.

Regarding the galacturonic acid content, the analysis revealed a high value of 66.3 ± 2.3%, indicating that the three-round extraction of phenolic compounds did not significantly solubilize polysaccharides such as pectin. Furthermore, the prior removal of phenolic compounds likely contributed to the purification of the starting material by reducing the competition between phenolics and pectin during the extraction process. This strategy favored the recovery of pectin with a higher purity and, consequently, an elevated galacturonic acid content.

From the FTIR spectra in Figure S2, the absorption peaks near 3600 cm^–1^ are observed to be related to the strong vibrations of hydroxyl (O–H) groups. The adsorption bands between 3000 and 2800 cm^–1^ are attributed to the stretching of the C–H bond of the CH_3_ and CH_2_ groups. Absorption bands below 1000 cm^–1^ correspond to the α and β anomeric configurations of pyranose and furanose rings.^46^ The degree of esterification of pectin was determined according to the method described by Naqash et al.,^47^ considering the ratio of esterified carboxyl methyl groups to the total number of carboxyl groups through the peak area at 1740 cm^–1^ (esterified methyl groups) and the peak area at 1616 cm^–1^ (nonesterified methyl groups). The degree of esterification was 66.38%, classifying pectin as highly methoxylated. Compared to the previous study,^32^ conventional extraction provided pectin with a degree of esterification of 84.59%. Thus, the previous extraction of phenolic compounds in three rounds may have affected the structure of pectin, decreasing the methoxyl content. The degree of esterification determines the functional properties and industrial applications of pectin, influencing aspects such as solubility, gel formation capacity, interaction with other compounds, and stability. Despite the moderate yield relative to other extraction technologies, the recovered pectin exhibited a relatively high degree of esterification, indicative of a preserved gelling capacity and structural integrity. This finding suggests that beyond the extraction yield, the quality of the obtained pectin is sufficient to underpin potential applications in food and pharmaceutical formulations.

Regarding the choice of the conventional heating extraction method, although emerging technologies such as microwaves,^48^ ultrasound,^49^ and pulsed electric field^50^ have emerged as highly efficient, accessibility, lower investment and maintenance costs, and ease of scaling make conventional heating extraction attractive for obtaining various compounds from plant matrices.

### 3.2 Biochar Characterization

#### 3.2.1 Scanning Electron Microscopy Analyses

Figure 2 presents the SEM micrographs before and after gallic acid adsorption at a 10 mg L^–1^ concentration. In Figure 2a, the surface appears fragmented and rough with irregular porous flakes. In contrast, Figure 2b shows a more granular and loosely aggregated structure, likely resulting from the prior removal of phenolic compounds and pectin. This may have caused an extensive loss of structural polysaccharides. Figure 2c reveals a more compact and fibrous network, featuring layered, leaf-like structures. The structure of the PP adsorbent in Figure 2d displays perfectly spherical, smooth, and nonporous particles, characteristic of synthetic polymeric resins. In contrast, Figure 2e depicts the heterogeneous and highly porous matrix of commercial AC, with needle- or column-like crystallites indicative of a high surface area and well-developed porosity.

**Figure 2:**
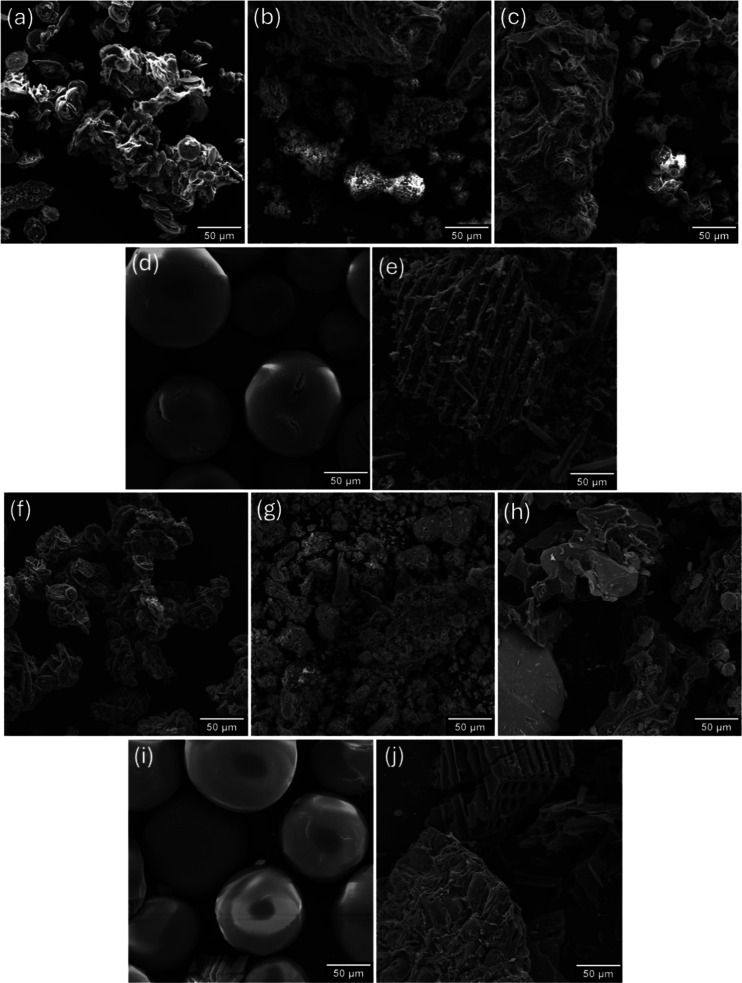
SEM micrographs—samples before adsorption: (a) CPB; (b) CPPB; (c) APB; (d) PP; (e) AC; samples after adsorption: (f) CPB; (g) CPPB; (h) APB; (i) PP; (j) AC.

Following adsorption, the CPB sample in Figure 2f exhibits a denser surface with visible agglomerates, likely corresponding to adsorbed gallic acid, suggesting successful surface interactions. In the CPPB sample (Figure 2g), gallic acid was adsorbed into microporous or irregular regions, forming a more consolidated structure with fewer visible pores. As shown in Figure 2h, the APB sample displays smoothed surfaces and infilled cavities, consistent with surface deposition or coating by gallic acid molecules.

The PP adsorbent (Figure 2i) exhibits slight surface deformation and minor deposits on the spheres, suggesting that adsorption occurs mainly via surface interaction or diffusion into probable internal pores. Finally, Figure 2j shows a pronounced coverage of porous structures in the AC sample, with some cavities appearing blocked and evident layered adsorption, aligning with a high gallic acid uptake. Gallic acid adsorption induced clear morphological changes across all adsorbents, particularly through surface coverage, pore blockage, and agglomerate formation. Apple pomace-derived biochars demonstrated more dynamic morphological transformations, likely due to greater surface accessibility and the availability of functional groups.

Elemental composition analysis revealed distinct chemical profiles among the adsorbents, as shown in Table 2. Biochar derived from apple pomace exhibited high carbon and oxygen contents, with phosphorus detected in substantial amounts, particularly in the APB sample (17.2 wt %) because of phosphoric acid activation. Notably, the progressive extraction of phenolic compounds and pectin led to an increase in carbon content, from 39.7 to 45.9 wt %, and a reduction in oxygen and inorganic elements, likely reflecting the removal of oxygenated compounds and soluble minerals. Silicon and aluminum were detected at low levels, possibly originating from environmental contamination or processing equipment. Commercial adsorbents displayed contrasting compositions: the PP sample consisted entirely of carbon, as expected for a synthetic polymer resin, while the AC sample showed a high carbon content (89.0 wt %) alongside notable levels of potassium, calcium, and magnesium, elements that may contribute to its high porosity and surface reactivity. The consistent presence of phosphorus across all biochars reinforces the role of H_3_PO_4_ in structural modification, enhancing the formation of phosphate-based functional groups that can facilitate adsorption mechanisms.

**Table 2:** Elemental Composition of Adsorbents

adsorbent	C (wt.%)	O (wt.%)	P (wt.%)	Si (wt.%)	Al (wt.%)	Na (wt.%)	K (wt.%)	Ca (wt.%)	Mg (wt.%)
CPB	41.9 ± 0.2	40.4 ± 0.2	12.3 ± 0.1	4.8 ± 0.1	0.6 ± 0.0	n.d.	n.d.	n.d.	n.d.
CPPB	45.9 ± 0.3	33.9 ± 0.2	13.7 ± 0.1	5.8 ± 0.1	0.4 ± 0.0	0.2 ± 0.0	n.d.	n.d.	n.d.
APB	39.7 ± 0.3	34.3 ± 0.2	17.2 ± 0.1	8.0 ± 0.1	0.2 ± 0.0	0.1 ± 0.0	n.d.	n.d.	n.d.
PP	100 ± 0.0	n.d.	n.d.	n.d.	n.d.	n.d.	n.d.	n.d.	n.d.
AC	89.0 ± 0.2	8.8 ± 0.2	0.7 ± 0.1	n.d.	n.d.	n.d.	1.0 ± 0.0	0.4 ± 0.0	0.1 ± 0.0

#### 3.2.2 BET and BJH Methods

Figure 3 exhibits the adsorption and desorption isotherms of the N_2_ gas for different adsorbents. The isotherms for the CPB sample were similar to type II,^51^ showing a probable presence of mesopores, micropores, and narrow crack pores on their surfaces, with irregular pore structures in these adsorbents. According to Figure 4, the pore distribution occurred at around 3.2 nm. Values between 2 and 50 nm, according to IUPAC, classify the adsorbent as mesoporous. Previous studies on biochar obtained from agricultural organic waste reported similar pore widths, varying between 3.6 ^4,52^ and 1 nm.^53^ Also, the pore size distribution of activated carbons encompasses microporous, mesoporous and macroporous structures.^54^ Therefore, as displayed in Figure 4, all adsorbents presented micro- and mesopores in their structures.

**Figure 3:**
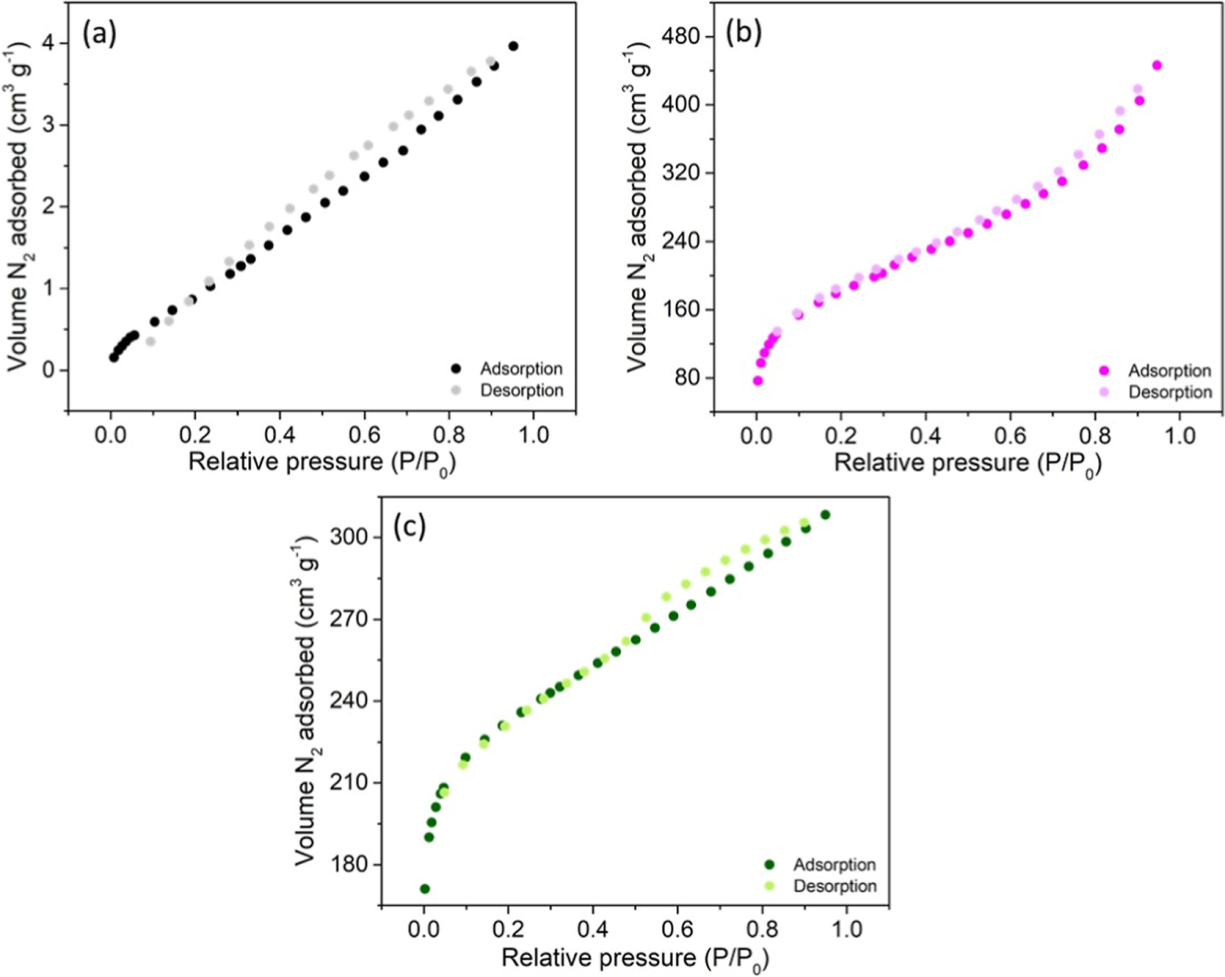
N_2_ adsorption–desorption isotherms for different biosorbents—(a) CPB; (b) PP; (c) AC.

**Figure 4:**
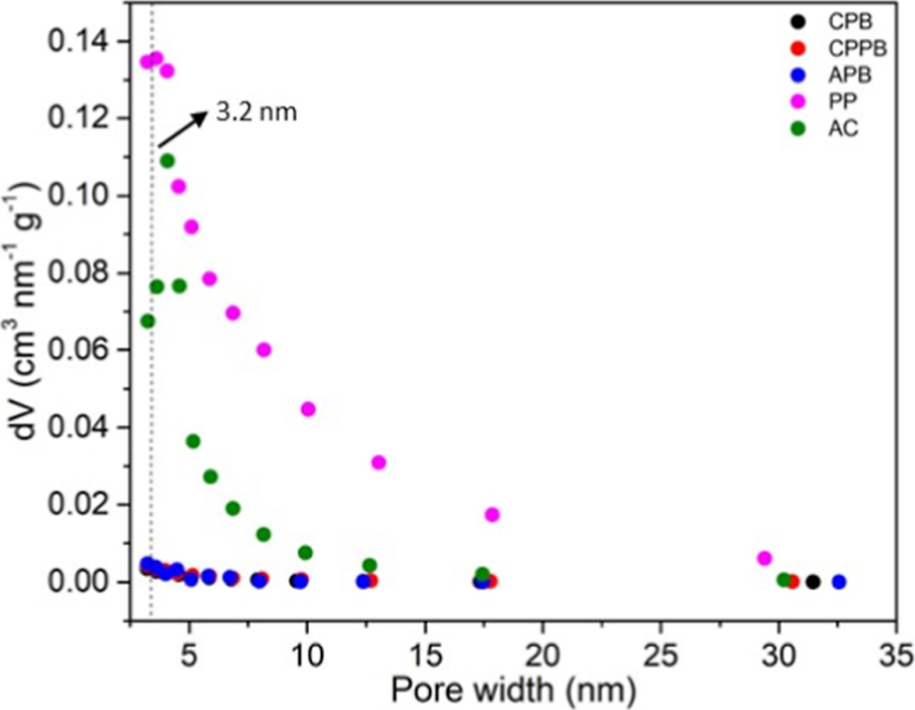
Pore size distribution analysis by the Barrett–Joyner–Halenda method.

Table 3 displays the BET surface area (*S*_0_), pore volume (*V*_p_), and mean pore diameter (*D*_p_) values of different adsorbents. AC and PP showed higher surface area and pore volume values than all apple pomace biochars, probably due to intensive activation processes and structured precursors in the synthesis process. Among the apple pomace byproduct adsorbents, CPPB exhibited the largest surface area of 5.66 m^2^ g^–1^ as well as the largest pore volume of 0.009 cm^3^ g^–1^. A larger surface area indicates that more active sites are available for interaction with target molecules. Similarly, a larger pore volume benefits the adsorption system by allowing for the diffusion of larger molecules.

**Table 3:** Textural Properties of Different Adsorbents

adsorbent	*S*_0_ (m^2^ g^–1^)	*V*_p_ (cm^3^ g^–1^)	*D*_p_ (nm)
CPB	3.85	0.005	5.16
CPPB	5.66	0.009	5.87
APB	4.88	0.005	4.36
PP	619.7	0.69	4.35
AC	741.2	0.48	2.61

#### 3.2.3 TGA and FTIR Analysis

The three-round extraction of phenolic compounds and sugars reduced the final residue mass available for biochar synthesis by 19.24 ± 0.04% compared to that of dry apple pomace. On the other hand, the final residue mass after the extraction of phenolic compounds, sugar, and pectin was reduced to 25.4 ± 2.0%. The higher residual mass, even with one more extraction step, probably occurred due to the interaction of citric acid with the functional groups of the cell wall, such as hydroxyl and carboxyl groups, leading to their retention in the structure. Likewise, the acid treatment may have promoted the cross-linking or restructuring of lignin and hemicelluloses, making them less soluble with the consequent higher final mass.

The TGA and DTG data of the apple pomace are shown in Figure 5a. From the masses at different degradation temperatures, the quantification of hemicellulose, cellulose, and lignin contents was calculated to be 30.7 ± 1.5%, 18.3 ± 0.1%, and 15.1 ± 0.8%, respectively. Variation in the composition of apple pomace may occur due to several factors: (1) apple cultivar—some varieties may have less insoluble fiber; (2) more aggressive methods of juice extraction in the industry may remove more cellulose; (3) pomace drying conditions may lead to the loss of organic matter; and (4) high fractions of pectin and hemicellulose indicate a reduced proportion of cellulose.

**Figure 5:**
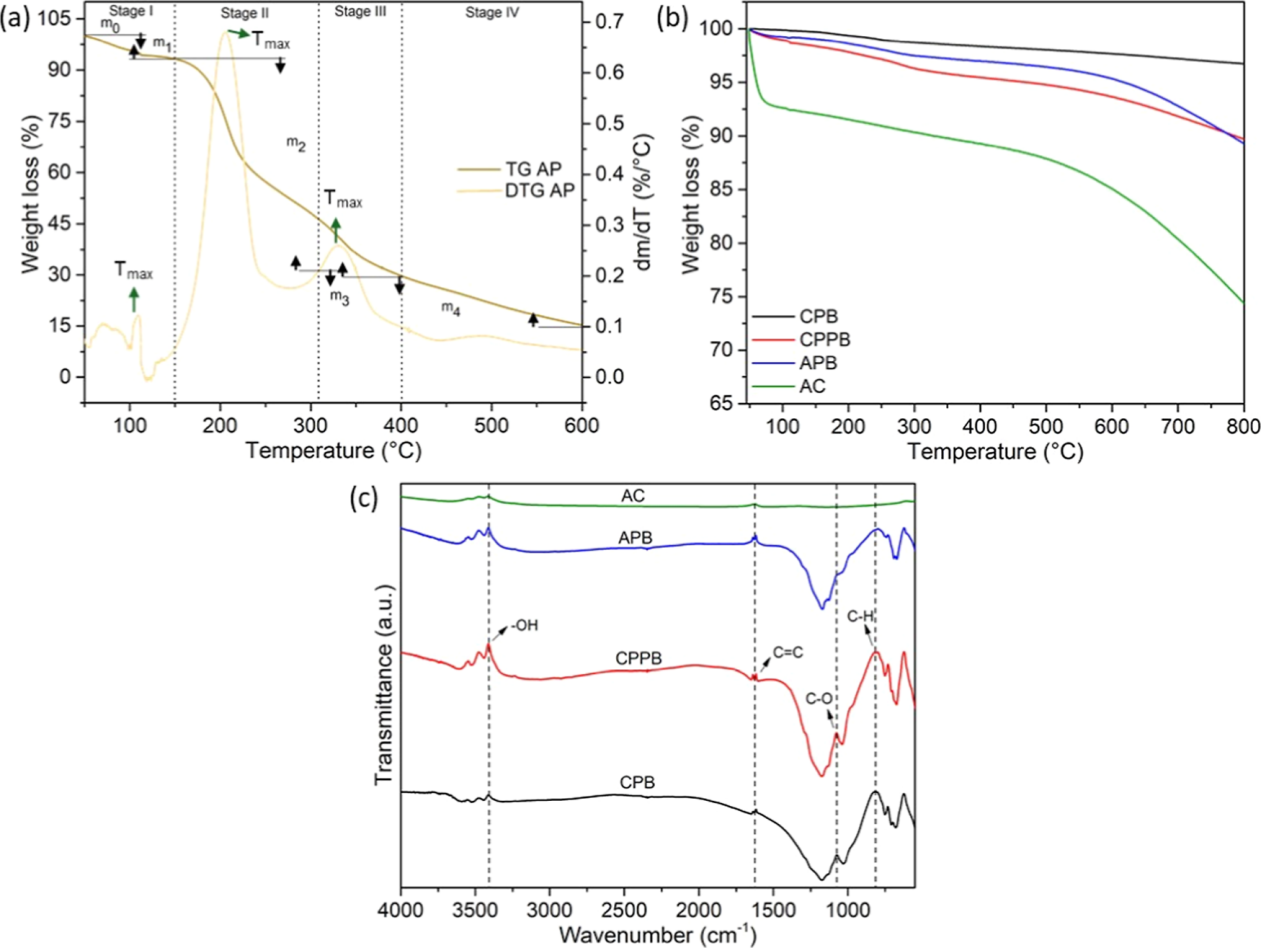
(a) TGA and DTG spectrum of apple pomace; (b) TGA spectra; and (c) FTIR spectra.

Regarding the stages of decomposition, stage I (up to 150 °C) presented a mass loss of up to 6.5%, removing moisture and light volatile organic compounds. Stage II is related to the decomposition of hemicellulose, presenting lower thermal resistance and less polymerization compared with the structures of cellulose and lignin. Stages III and IV are associated with the degradation of cellulose and lignin, respectively. The sharp weight loss between 300 and 550 °C is due to the breakdown of cellulose,^55^ increasing from 51.5 to 81.5%. Up to 300 °C, the thermal decomposition process forms carbon dioxide, carbon monoxide, and other carbonaceous gases. Beyond this temperature, liquid-phase compounds are generated.^56^ A gradual breakdown of lignin occurs above 550 °C,^57^ being the thermally resistant section of the plant matrix that produces charcoal due to the aromatic rings in its structure. The separation of the peaks in the DTG curve highlighted the distinction between hemicellulose and cellulose, demonstrating that these components are structurally differentiated in apple pomace.

Figure 5b displays the TGA data for samples CPB, CPPB, APB, and AC. The first region below 200 °C referred to weight loss due to the release of physically adsorbed water and volatile moisture. There were weight losses of 0.83, 1.66, 2.40, and 8.50% for samples CPB, CPPB, APB, and AC, respectively. Sample AC showed a high amount of volatile material and moisture compared with the other samples. Similarly, its weight loss at 700 °C was 25.84%, a value much higher than 3.34, 10.60, and 10.50%, for samples CPB, CPPB, and APB. Although the acid treatment with H_3_PO_4_ for biochar activation leads to the formation of vulnerable pores and void sites that are easily thermally attacked,^58^ the treatment for the activation of commercial activated carbon may have been more aggressive, leading to greater degradation at lower temperatures than the other samples. In general, the biochar samples were stable in an inert N_2_ environment with a maximum degradation of 10.60%.

According to Figure 5c, the FTIR analysis revealed peaks near 3400 cm^–1^, corresponding to the – OH stretching vibration, highlighting the hydroxyl groups. These groups play an important role in the adsorption of compounds due to the formation of hydrogen bonds with the adsorbate.^59^ The peaks within the range of 800–1700 cm^–1^ are attributed to aromatic functional groups such as C=C, C=O, and C–OH that characterize the presence of aromatic structures in biochar.^60^ Xi et al.^61^ reported that aromatic fractions can exhibit π–π interactions, contributing to the adsorption of organic contaminants that are aromatic in nature. A small peak near 1650 cm^–1^ can refer to the C=C stretching of aromatic components.^62^ A peak near 1063 cm^–1^ was related to C–O bonds in ethers, esters, and carbonates.^63^ Although the final extraction residues were different, all biochars from apple pomace showed little change in the peaks, probably due to the same synthesis conditions. Additionally, the appearance of the peak near 805 cm^–1^ indicated the presence of C–H stretching vibrations in the aromatic rings.^64,65^

### 3.3 Adsorption Tests

Adsorption tests aim to elucidate the adsorption capacity of a biochar. Figure 6 highlights the adsorption tests of gallic acid at various concentrations by using different adsorbents. According to Figure 6a, the CPPB sample stood out with an adsorption efficiency of 89.93 ± 0.87% compared to the other biosorbents, being inferior only to commercial AC (99.51 ± 0.58%), considering a gallic acid concentration of 10 mg L^–1^. The adsorption performance of the CPPB sample that highlights the quality of the biochar in terms of functional groups, porosity, and surface reactivity was superior, making it comparable to commercial AC. The commercial biosorbent PP presented an adsorption efficiency of 31.4 ± 0.6%. The difference between the two commercial biosorbents is probably due to oxygenated functional groups on the AC surface, facilitating interactions with gallic acid molecules. Additional treatments of PP could increase its surface functionality for gallic acid. Similarly, Barroso et al.^53^ reported a 15% lower efficiency of PP compared to commercial AC in the adsorption of cyanidin-3-glucoside using an adsorbent concentration of 1 g L^–1^, at 25 °C and 100 rpm for 24 h.

**Figure 6:**
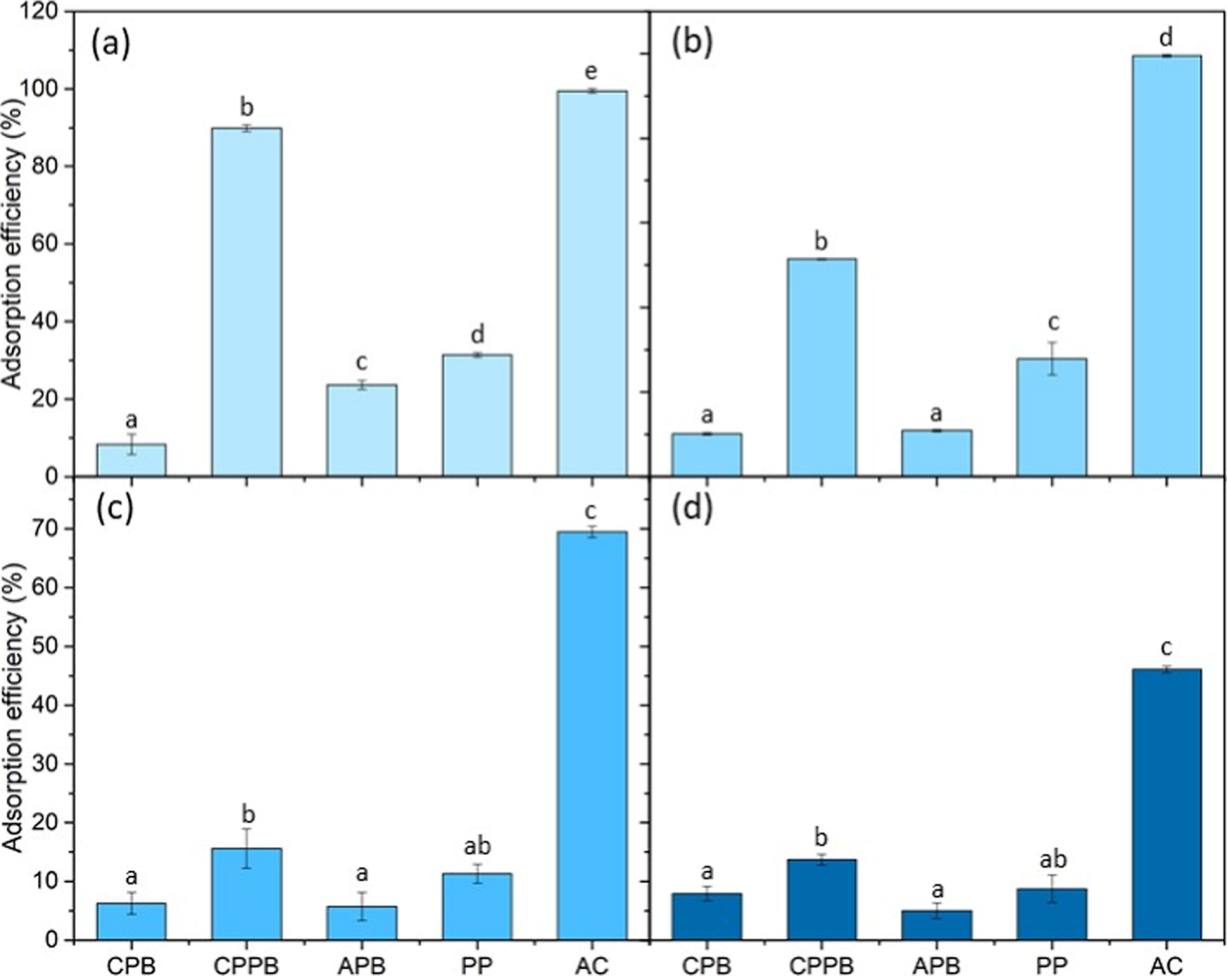
Efficiency of gallic acid adsorption tests at different concentrations—(a) 10; (b) 50; (c) 250; and (d) 500 mg L^–1^.

The high efficiency of CPPB compared to CPB (8.4 ± 2.6%) and APB (23.65 ± 1.15%) samples suggested that the sequential extraction of phenolic compounds with ethyl alcohol and subsequently pectin with a citric acid solution resulted in a more porous material with a larger surface area, favoring the adsorption of gallic acid. The preservation of acidic groups such as carboxyls, carbonyls, and hydroxyls on the biochar surface caused by citric acid increased the interaction with the adsorbate via hydrogen bonds and electrostatic interactions. In another proposed hypothesis, the multiple extractions using ethanol and citric acid reduced the amount of volatile compounds and organic residues with the consequent unclogging of pores that would limit the adsorption capacity. Besides, the CPB sample may have presented only part of the free pores for adsorption, containing residual pectin and limiting the formation of efficient micropores for adsorption. Furthermore, the APB sample may have had more residual organic compounds, resulting in fewer pores available to facilitate adsorption.

Comparing Figure 6b–d, the adsorption efficiency slightly decreased for most of the adsorbents, with the exception of AC. Its efficiency reduced from 99.52 ± 0.33% to 46.08 ± 0.56%, considering the gallic acid concentrations of 50 and 500 mg L^–1^, respectively. Although the adsorption efficiency decreased with the increasing adsorbate concentration, the amount of gallic acid adsorbed increased. This behavior occurred due to the greater number of molecules available in the more concentrated solution to interact with the adsorbent. On the other hand, with the increasing gallic acid concentration, more molecules try to occupy the same active sites, causing a gradual saturation of the active sites. Likewise, the maximum adsorption capacity of biochar is reached, resulting in a lower efficiency. However, since the initial concentration was higher, the total amount of gallic acid retained was still greater.

### 3.4 Adsorption Kinetics and Reuse Test

Kinetic analysis aims to determine equilibrium, estimate the adsorption rate, and understand process stages, including diffusion and convection. Various models are tested to identify the most suitable one, considering their ability to describe adsorption based on previous studies of similar systems. The pseudo-first-order kinetic model is effective in the initial stage of adsorption but exhibits limitations over extended contact times. It describes the sorption rate as dependent on the driving force associated with unoccupied adsorption sites, decreasing as adsorption progresses.^66^ In contrast, the pseudo-second-order model predicts the adsorption behavior throughout the entire process, suggesting that chemisorption is the rate-limiting step. However, this model does not account for diffusion effects, potentially influencing the accuracy of adsorption mechanism characterization.^67^

The Elovich kinetic model initially described the chemisorption of gases onto solid surfaces. However, its application has been extended to liquid-phase sorption, particularly in systems where the adsorbent surface is heterogeneous and desorption is negligible.^68^ Furthermore, this model has been employed to assess the mass and surface diffusion processes and estimate the activation and deactivation energies associated with adsorption.^69^ Additionally, valence forces can occur in interactions between the adsorbent and adsorbate.^70^ Finally, the Avrami model, originally developed to describe the kinetics of phase transitions, can be adapted to study the adsorption processes. It allows investigating mechanisms considering the interplay of diffusion, surface reactions on the adsorbent, and pore filling.^71^

Figure 7a shows the adsorption kinetics of gallic acid at a concentration of 10 mg L^–1^, pH 3.5, and 25 °C, considering the CPPB sample and different model adjustments. After 1 min of adsorption kinetics, more than 50% of gallic acid had been adsorbed by the biochar. After 15 min of the adsorption process, the adsorbed amount remained constant, reaching adsorptive equilibrium with a *q*_e_ value of 7.7 ± 0.1 mg g^–1^ of biochar. The Avrami model presented the best fit to the experimental data, with an adjusted *R* ^2^ value of 0.9953, as shown in Table 4. This model has stood out in several applications such as the adsorption of gas capture on mesoporous materials^72^ and metals in wastewater.^73^ It considers complex processes involving cooperative adsorption and reorganization of the adsorbent surface. The Avrami exponent (*n*_A_) indicates variations in the mechanism of the adsorption process.^74^ The fractional value of 0.37 suggests the complexity of the reaction mechanism or the simultaneous occurrence of multiple reaction pathways.^75^

**Figure 7:**
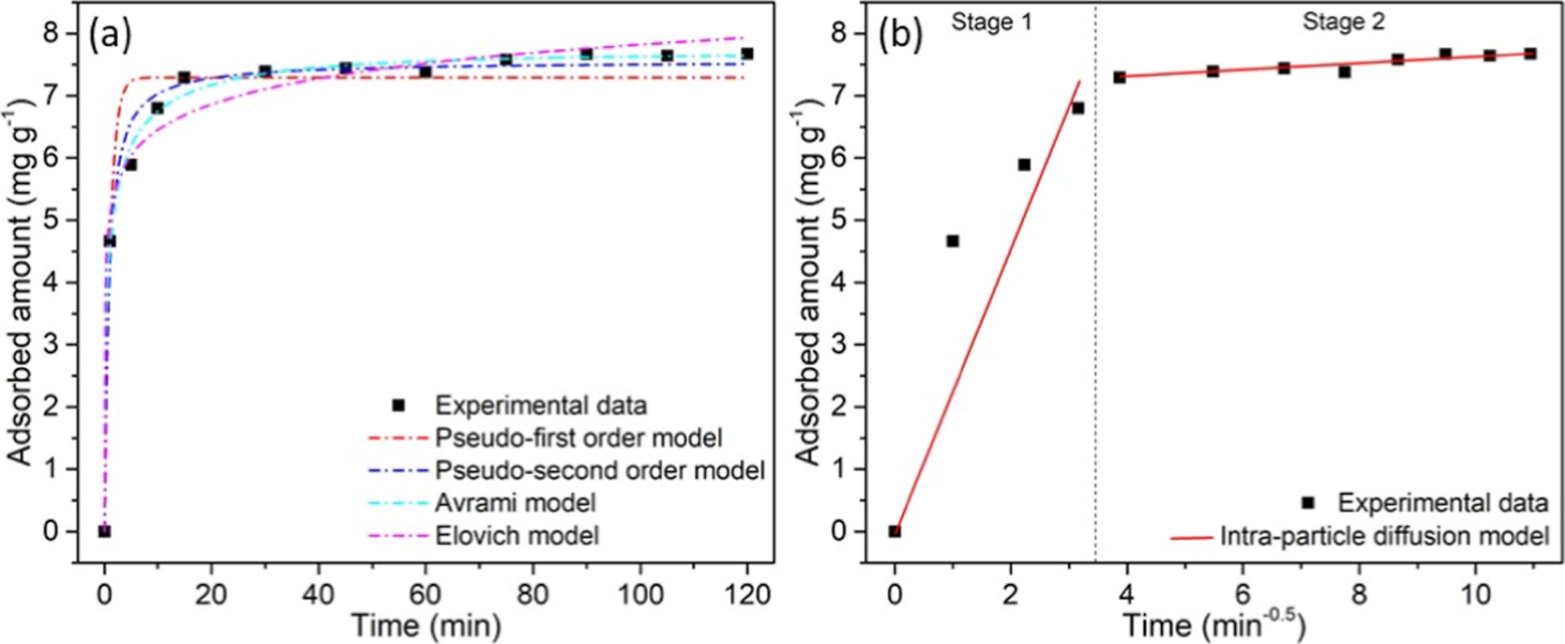
Adsorption kinetics of the CPPB sample—(a) models of pseudo-first-order, pseudo-second-order, Avrami, and Elovich; (b) intraparticle diffusion model.

**Table 4:** Kinetic Parameters of Different Models for Adsorption of Gallic Acid with a Concentration of 10 mg L^–1^ in the CPPB Sample

model/system	CPPB sample
experimental data	*q*_e_ = 7.7
pseudo-first-order	*q*_e_ = 7.3 ± 0.2 mg g^–1^
	*k*_1_ = 0.97 min^–1^
	*R* ^2^ = 0.9524
	adjusted *R* ^2^ = 0.9476
pseudo-second-order	*q*_e_ = 7.6 ± 0.1 mg g^–1^
	*k*_2_ = 0.18 g min^–1^ mg^–1^
	*R* ^2^ = 0.9865
	adjusted *R* ^2^ = 0.9852
Avrami	*q*_e_ = 7.7 ± 0.1 mg g^–1^
	*k*_A_ = 0.91 min^–1^
	*n*_A_ = 0.37
	*R*_2_ = 0.9962
	adjusted *R* ^2^ = 0.9953
Elovich	α = 2924 mg g^–1^ min^–1^
	β = 1.67 g mg^–1^
	*R* ^2^ = 0.9832
	adjusted *R* ^2^ = 0.9815
intraparticle model-stage 1	*k*_id1_ = 2.46 mg g^–1^ min^–0.5^
	*C*_1_ = 0 mg g^–1^
	*R* ^2^ = 0.9420
	adjusted *R* ^2^ = 0.9227
intraparticle model-stage 2	*k*_id2_ = 0.06 mg g^–1^ min^–0.5^
	*C*_2_ = 7.05 mg g^–1^
	*R* ^2^ = 0.8652
	adjusted *R* ^2^ = 0.8427

Figure 7b displays the intraparticle diffusion model for the adsorption of gallic acid on the CPPB sample. The multilinearity suggested that intraparticle diffusion did not solely govern the adsorption rate of gallic acid. According to Table 4, the boundary layer thickness (C) for stage 1 presented a value of 0, while that for stage 2 presented 7.05 mg g^–1^. The intraparticle diffusion constant presented a higher value for stage 1 of 2.46 mg g^–1^ min^–0.5^, compared to stage 2 (*k*_id2_ = 0.06 mg g^–1^ min^–0.5^), suggesting that gallic acid molecules are rapidly adsorbed on the external surface of CPPB. Once the external surface of the biochar is saturated with gallic acid, the molecules penetrate into the pores, leading to adsorption within the particles at the active sites on the internal surface of the adsorbent. Monteagudo et al.^76^ reported a similar intraparticle diffusion mechanism for CO_2_ adsorption in KOH-activated olive pomace biochar at a biochar/KOH ratio of 1:0.5. The multilinearity in the study provided two stages: (1) diffusion of the gas through the air to the external surface of the biochar; (2) adsorption corresponding to intraparticle diffusion of the gas inside the pores of the biochar. Likewise, the authors reported a higher value of the intraparticle diffusion constant in the first stage compared with the second stage.

Figure 8 highlights the reuse capacity of the CPPB sample considering 7 cycles of use. There was a slight loss of adsorption capacity as the number of cycles increased. The adsorption capacity in the first cycle was 8.21 mg g^–1^ compared to 7.15 mg g^–1^ in the seventh cycle, demonstrating a 12.9% loss of capacity. This drop in efficiency does not affect the application of the biosorbent and highlights its excellent performance for the adsorption of gallic acid. A small loss of material may have occurred between cycles due to erosion of the biochar matrix, reducing the amount of available active sites. Likewise, desorption with the concentrated ethanol solution may have solubilized residual organic compounds from the biochar, removing less stable fractions from the matrix. Although the ethanol/water ratio of 1:1 has been reported as having the highest desorption efficiency (91%) of polyphenols from porous activated carbon,^77^ in this study, the 70% v/v ethanol solution presented a maximum desorption efficiency of 94.9% for the first cycle. Dipolar solvents, like ethanol, can minimize hydrophobic interactions between the adsorbent and adsorbate molecules, facilitating desorption.^78^ According to the method described by Galanakis et al.,^79^ the recovery of polyphenols is directly linked to their solubility, varying according to the solvent. Solvents of intermediate polarity are more effective in the extraction of these compounds, while highly polar solvents, such as water, or less polar solvents, such as ethyl acetate and acetone, present lower efficiency in this process. In addition, other factors influencing desorption include surface properties (functional groups), thermal stability, and chemical stability of the adsorbent.^80^

**Figure 8:**
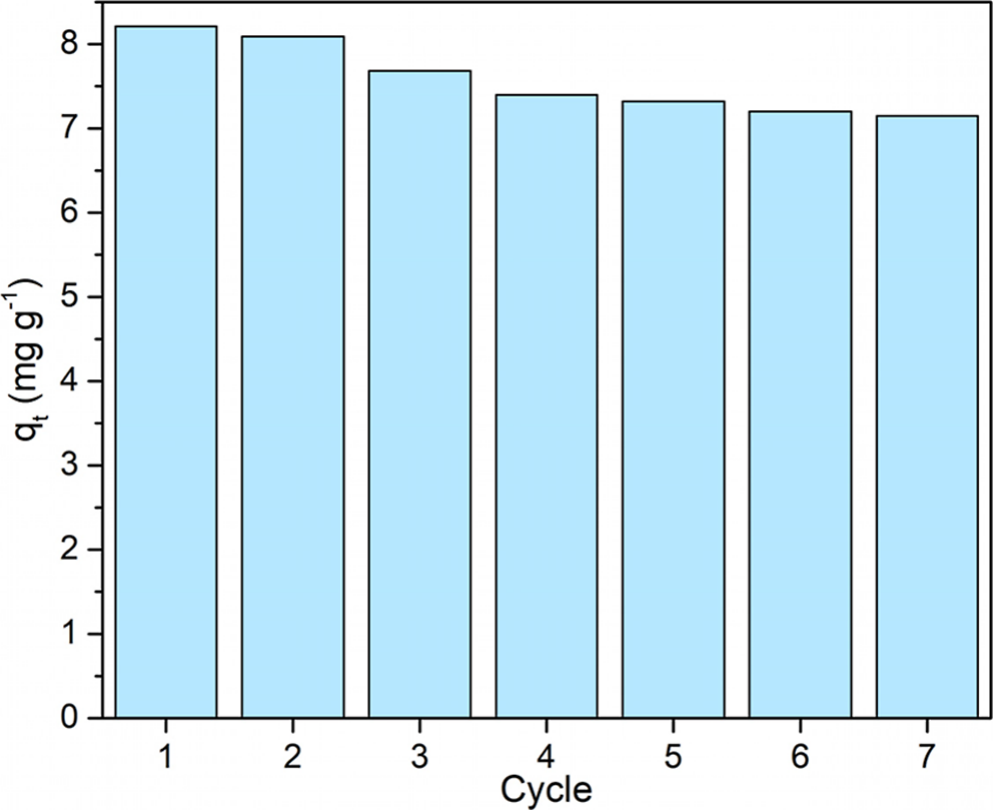
CPPB sample reuse cycles in gallic acid adsorption.

### 3.5 Proposed Adsorption Mechanism

The efficiency of gallic acid adsorption onto biochar is governed by multiple factors, including the molecular structure of polyphenols, the physicochemical characteristics of the adsorbent, and the operational conditions employed during the process.^81^ FTIR analyses and SEM micrographs revealed a substantial surface area and the presence of key functional groups such as hydroxyl (O–H), ether (C–O), alkene (C=C), alkyl (C–H), and acidic moieties. These surface features enhance the interaction between gallic acid molecules and the biochar surface. The adsorption process is largely driven by the electrostatic attraction between the ionized gallic acid species and the negatively charged sites on the adsorbent. Furthermore, π–π interactions between the aromatic rings of gallic acid and the graphitic domains of activated carbon play a significant role in stabilizing the adsorbed species.^82^ The presence of electron-withdrawing substituents on polyphenolic structures can further reinforce these π–π interactions by diminishing electrostatic repulsion, thereby increasing the affinity toward the adsorbent.^83^ A similar mechanism may have occurred for apple pomace biochar samples. The adsorption was likely driven by interactions between the adsorbent and the oxygen atoms as well as the aromatic rings of gallic acid. The removal of this compound is associated with the formation of hydrogen bonds involving carbonyl and hydroxyl groups on the adsorbent surface and the nonbonding electron pairs of oxygen atoms and hydroxyl groups within the adsorbent.^81^ Based on the interaction mechanisms described, this study has strong potential for application in treating industrial effluents containing phenolic compounds, such as gallic acid, common in waste from the food, pharmaceutical, and beverage industries. The use of biochar derived from apple pomace as a sustainable adsorbent can represent a low-cost and high-efficiency alternative for the removal of organic contaminants, contributing to environmental solutions aligned with the principles of the circular economy.

### 3.6 Green Assessment of the Apple Pomace Biorefinery—Path2Green Metric

Operating the industrial process in continuous mode positively influences the scale-up of the phenolic compound, sugar, and pectin extraction steps, being one of the 12 principles of the Path2Green metric with regard to environmental, social, and economic impacts. However, considering conventional batch heating extraction, the Path2Green metric demonstrated a negative effect on the process scale-up according to the score −1. As reported in Figure 9, the TPC, sugar, and pectin extraction processes provided a score of 0.665. A score value close to 1 indicates a greener and more sustainable extraction process.

**Figure 9:**
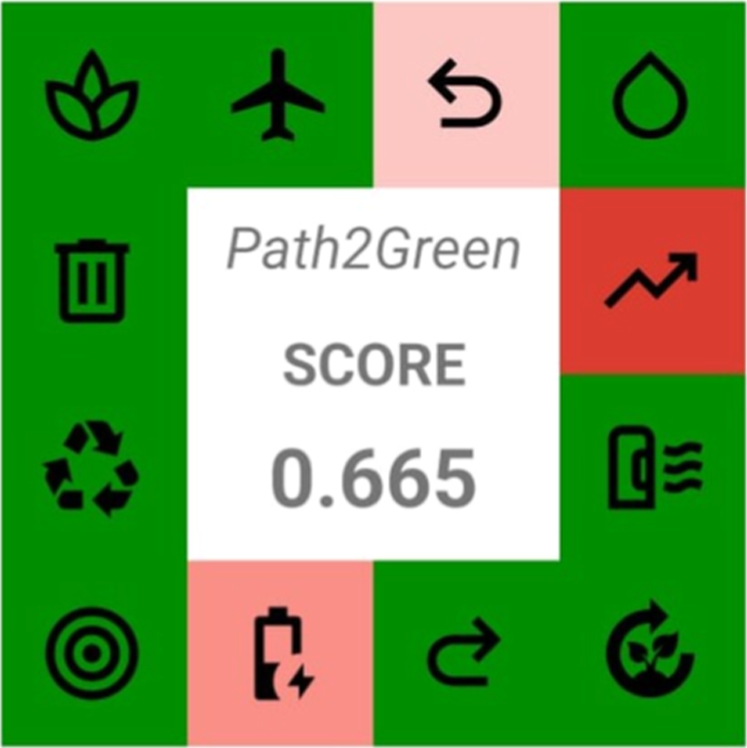
Scores of the extraction process of phenolic compounds, sugar, and pectin.

Regarding the minimization of pretreatment, apple pomace required only physical treatment for extraction, assigning a score of −0.2 in the metric. The principle governing biomass transport assumed a distance of 1 km between the biomass source and the extraction facility. The expectation is that a zero-waste biorefinery for apple pomace would be constructed adjacent to a juice processing plant, thereby eliminating the costs and environmental impacts associated with transport. For the waste principle, 0% residue generation was assumed, given the proper management of the citric acid solution after pectin precipitation and the recycling of ethanol.

For the energy principle, high dependence and use of renewable energy (hydroelectric sources) were considered, presenting a score of −0.5. Since the process used a byproduct for extraction, organic and nonhazardous solvents (ethanol and citric acid), did not require purification of the extracts, and presented complete valorization of the biomass, a score of +1 was assigned. Extraction processes that require toxic solvents such as hexane and multiple post-treatment steps result in scores close to 0 and even negative.^84^ Furthermore, the readiness for use of the extracts, application in several fields, such as the food, pharmaceutical, and cosmetic industries, in addition to using nonvirgin raw material and minimal waste generation, also resulted in a score of +1. Thus, although the extraction process is aligned with the 12 principles of the Path2Green metric, continuous flow extraction studies can improve the score and provide insights for large-scale applications.

## 4 Conclusion

A comprehensive biorefinery approach applied to apple pomace yielded extracts rich in sugars and phenolic compounds, in addition to producing biochar and enabling pectin precipitation. The three-round extraction process demonstrated that the plant matrix retained bioactive compounds even after successive recoveries, although at lower concentrations. This strategy maximized the utilization of valuable constituents within the agro-industrial byproduct, offering sustainable solutions for the agri-food sector while reducing the environmental impact associated with waste disposal. Furthermore, the final residue obtained after the extraction of phenolics and pectin exhibited the highest gallic acid adsorption efficiency (89.93 ± 0.87%) compared to biochar from apple pomace and biochar from the residue after the extraction of phenolic compounds, demonstrating that even extensively processed biomass can serve as a highly effective biosorbent. In this context, the study proposes viable alternatives for organic solid waste management and the valorization of natural biocompounds through sustainable extraction technologies, as evaluated using the Path2Green metric. The application of this framework highlighted the potential of waste-derived valorization pathways to align with green chemistry principles and the objectives of a circular economy. Additionally, these findings emphasize the potential of biowaste-derived materials in high-value applications, bridging the gap between waste management and sustainable functional materials.

## Supplementary Material



## Data Availability

The data supporting this article have been included as part of the Supporting Information.

## Notes

Procedures – chemicals and raw material, biochar synthesis, characterization of the extracts (total phenolic compounds, sugars, organic acids, inhibitors, antioxidant activity, and galacturonic acid), Path2Gren metric. Figures – chromatogram of different compounds in the extracts, and FTIR spectra of pectin extracted from apple pomace after the extraction of phenolic compounds and sugars (PDF)
