# Supplementary material for: Transforming Waste into Value: A Sustainable Zero-Waste Biorefinery for Biochar Production and Gallic Acid Adsorption from Apple Pomace
Source: ACS Omega. 2025 Oct 17;10(42):50252–66. doi: 10.1021/acsomega.5c06957 (PMC12573183; doi:10.1021/acsomega.5c06957)
Supplement: Supplementary file 1 [file ao5c06957_si_001.pdf]

SUPPLEMENTARY MATERIAL

Transforming waste into value: a sustainable zero-waste biorefinery for biochar production and gallic acid adsorption from apple pomace

Josiel Martins Costa[1*], Leda Maria Saragiotto Colpini[2], Tânia Forster-Carneiro[1*]

[1] *Faculdade de Engenharia de Alimentos (FEA), Universidade Estadual de Campinas (UNICAMP), Rua Monteiro Lobato, 80, 13083-862, Campinas, São Paulo, Brazil*

[2] *Graduate Program in Technology and Environmental Engineering, Federal University of Parana (UFPR), Rua Pioneiro, 2153, 85953-128, Palotina, Paraná, Brazil*

* Corresponding author: josiel.martins.costa@gmail.com, taniafc@unicamp.br

1. Procedures

1.1. Chemicals and raw material

Apple pomace was supplied by Oriente Consultoria e Representações LTDA (Videira, SC, Brazil). Citric acid (99.5%) was obtained from Synth (Diadema, Brazil). The following analytical-grade chemicals were purchased from Sigma Aldrich (Steinheim, Germany): Trolox (6-hydroxy-2,5,7,8-tetramethylchroman-2-carboxylic acid, 97%), m-hydroxyphenyl (85%), TPTZ (2,4,6-tripyridyl-s-triazine, ≥99%), and D-(+)-galacturonic acid monohydrate (≥97%). Sodium tetraborate (99.5%), sodium carbonate (99.5%), ferric chloride (97%), sodium acetate (99%), Folin–Ciocalteu reagent, and sodium hydroxide (97%) were obtained from Dinamica (São Paulo, Brazil). PoraPak Rxn (PP) was sourced from Waters Corporation (Milford, MA, USA), and activated carbon was acquired from Ecibra (São Paulo, Brazil). All solutions were prepared using distilled water (Milli-Q Direct 8, Merck, HE, Germany).

1.2. Biochar synthesis

The dried samples of the final residues after sequential extractions of phenolic compounds and pectin and apple pomace were placed in individual mortars and calcined at 400 °C in a muffle furnace for 3 h. After calcination and cooling of the samples in a desiccator to room temperature, the activation step occurred using H_3PO_4 85% in a 10:1 v/w ratio of biochar. The acid in contact with biochar was macerated with the pestle for 5 min. The liquid mixture was left to stand for 4 h. After that, the samples were dried in the oven at 105 °C for 12 h to remove residual water. The samples were activated for 3 h in the muffle furnace at 400 °C. After the samples cooled to room temperature, the biochars were washed with a 2% w/v $NaHCO_3$ solution using vacuum filtration until the pH of the solution was neutralized. The samples were dried in an oven at 105 °C for 2 h

and then stored in a desiccator. The biochar mass was determined by the weight of the filter paper before washing and after adding the samples and drying in the oven. The biochar production yield considered the mass of final dry residue and the mass of biochar.

1.3. Characterization of the extracts

1.3.1. Determination of total phenolic compounds

A 300 µL aliquot of the diluted sample was mixed with 300 µL of the Folin-Ciocalteu reagent and 2400 µL of 5% (w/v) sodium carbonate solution. The mixture was then placed in a dark environment for 20 min. Afterwards, the absorbance of the solution was measured at 760 nm against a blank using a UV-vis spectrophotometer (Bel, UV-M51, SP, Brazil). A calibration curve was established using gallic acid as a standard, with concentrations ranging from 10 to 50 µg mL^{-1} and R^2 = 0.99. The total phenolic compounds of the extracts were quantified in milligrams of gallic acid equivalents per gram of apple pomace (mg GAE g^{-1}).

1.3.2. Determination of sugars, organic acids, inhibitors, and galacturonic acid

The concentration of glucose (0–1 g L^{-1}; R^2 = 0.99), fructose (0–1 g L^{-1}; R^2 = 0.99), acetic acid (0–2 g L^{-1}; R^2 = 0.99), and 5-hydroxymethylfurfural (0–1 g L^{-1}; R^2 = 0.98) were determined from the calibration curves of each standard. The analysis was performed in triplicate and the results were expressed in mg g^{-1} of apple pomace.

The galacturonic acid concentration was determined from the calibration curve (0–1 g L^{-1}; R^2 = 0.99) and the result was expressed in %. Briefly, 2.5 mL of 2M TFA was added to 30 mg of dry pectin in a screw-capped test tube and heated in a water bath at 95 °C for 60 min. Then, the

hydrolysate was diluted with 5 mL of 1 M NaOH and adjusted to pH 6 with 0.1 M NaOH. After this, the extract was diluted in water, followed by filtration with a 0.22 μm filter.

1.3.3. Determination of antioxidant capacity

A 10 mmol L^{-1} TPTZ solution was prepared using 40 mmol L^{-1} HCl as the solvent. Both the sodium acetate buffer and the ferric chloride solution were prepared with distilled water. The FRAP reagent was freshly prepared by mixing 0.3 M sodium acetate buffer (pH 3.6), 10 mmol L^{-1} TPTZ, and 20 mmol L^{-1} ferric chloride in a volumetric ratio of 10:1:1 (v/v/v). For the assay, 90 μL of the diluted extract were transferred to a test tube, followed by the addition of 2.7 mL of the FRAP reagent and 270 μL of distilled water. The mixtures were vortexed and incubated in a thermostatic water bath at 37 °C for 30 min (TECNAL, model TE-2005, SP, Brazil). After incubation, absorbance was measured at 595 nm using a UV-vis spectrophotometer (Bel UV-M51, SP, Brazil). Antioxidant activity was quantified based on a Trolox calibration curve (5–400 μM, $R^2 = 0.99$), and results were expressed as micromoles of Trolox equivalents (TE) per gram of apple pomace.

1.4. Path2Green metric

The metric is based on 12 green extraction principles, considering factors such as: 1-Biomass; 2-Transportation; 3-Pre-treatment; 4-Solvents; 5-Scale, 6-Purification, 7-Yield, 8-Post-treatment; 9-Energy; 10-Application; 11-Reuse and 12-Waste Management. Each principle is scored between -1 and +1. A score closer to +1 indicates better adherence to the green principle. The assessment was carried out using the Path2Green mobile application, providing a pictogram

of the metric. The pictogram indicates the final score and performance of the extraction process, allowing the process to be improved in terms of sustainability.

Supplementary figures

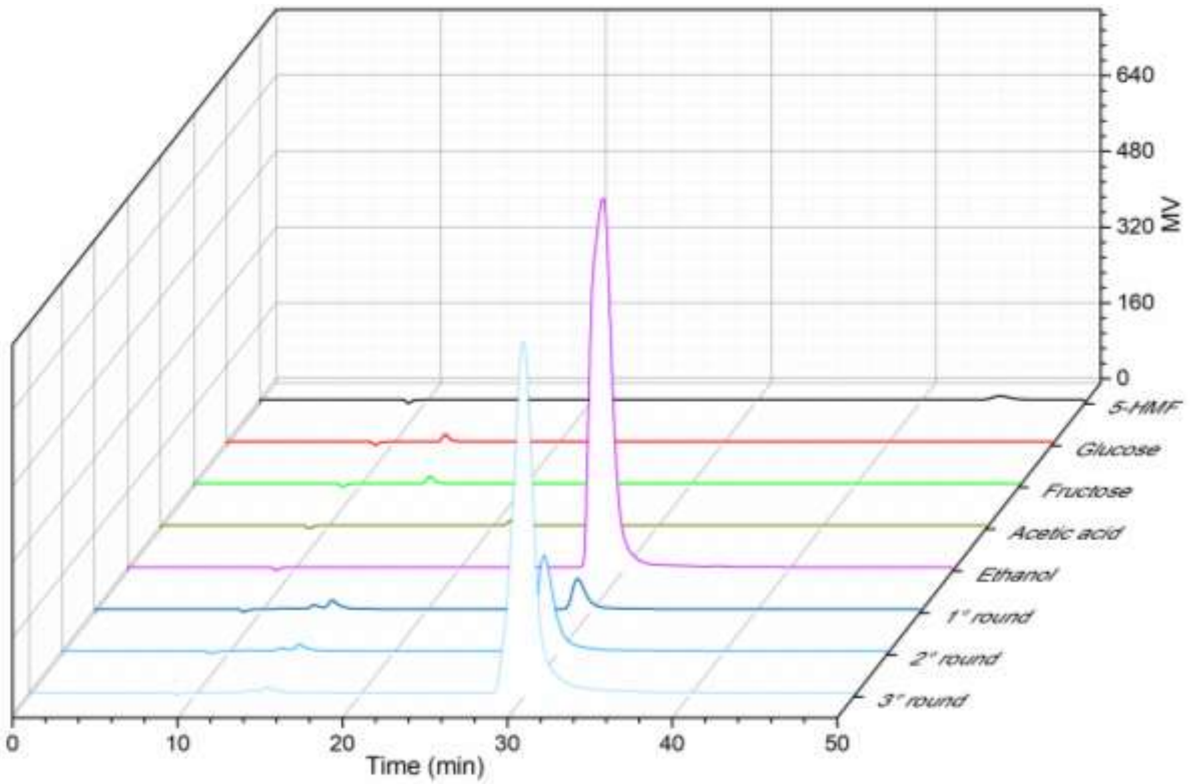

Figure S1. Chromatogram of different compounds in the extracts.

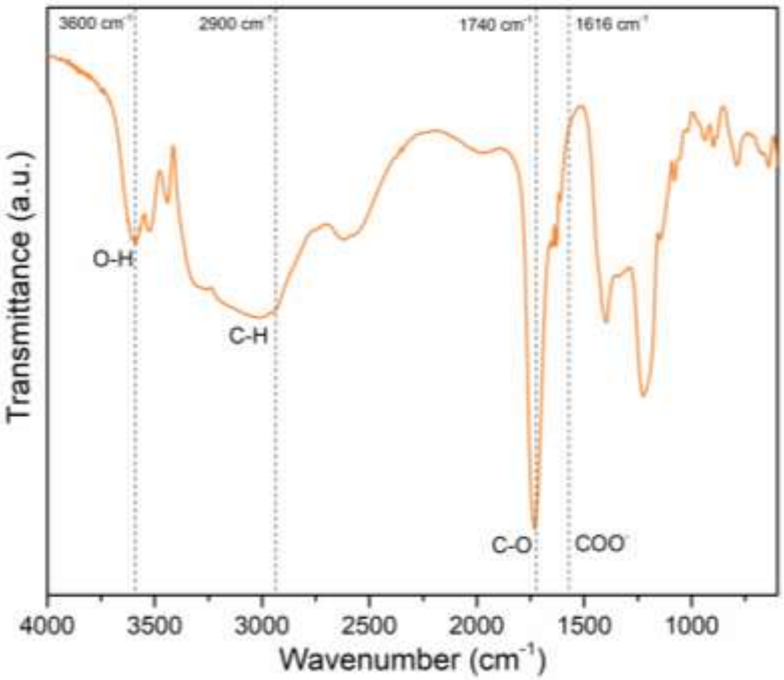

Figure S2. FTIR spectra of pectin extracted from apple pomace after extraction of phenolic compounds and sugars.
